# nm23 protein expression in metastatic and non-metastatic tongue squamous cell carcinoma

**DOI:** 10.1016/S1808-8694(15)30568-1

**Published:** 2015-10-19

**Authors:** Éricka Janine Dantas da Silveira, Márcio Campos Oliveira, Maria de Lourdes Silva Arruda de Morais, Lélia Maria Guedes Queiroz, Antonio de Lisboa Lopes Costa

**Affiliations:** 1 Doctorate in oral pathology, UFRN. Adjunct professor of the Universidade do Estado Rio Grande do Norte.; 2 Doctorate in oral pathology, UFRN. Adjunct professor, Dentistry Department, Universidade Estadual de Feira de Santana.; 3 Master's degree in oral pathology; doctoral student in Health Sciences, UFRN. Responsible for the Dentristry Unit of the Dr. Luiz Antonio Hospital.; 4 Doctorate in oral pathology, USP. Adjunct professor of oral pathology, UFRN, and of the graduate program in oral pathology, Universidade Federal do Rio Grande do Norte, Natal/RN.; 5 Doctorate in oral pathology, USP. Adjunct professor of oral pathology, UFRN, and of the graduate program in oral pathology, Universidade Federal do Rio Grande do Norte, Natal/RN. Universidade Federal do Rio Grande do Norte.

**Keywords:** tongue squamous cell carcinoma, immunohistochemistry, nm23 protein

## Abstract

Oral squamous cells carcinoma (OSCC) shows unfavorable prognosis due to its invasion potential around the neighboring tissues and the elevated incidence of metastasis.

**Aim:**

the present paper aims at analyzing the immunohistochemical expression of the nm23 protein in metastatic and non-metastatic SCCs of tongue.

**Methods:**

the immuno-expression to the nm23-hl protein was diagnosed in 35 tongue SCC (15 of which exhibiting metastasis). Nm23-hl immuno-scores were assigned as follow: score 0 = absent, 1 = focal and 2 = diffuse expression.

**Results:**

The Fisher's exact test was performed and there was no statistical difference between the nm23-hl immuno-scores and the tongue SCCs studied cases (p=0.365), although 66.7% of metastatic cases presented negative nm23-hl expression.

**Conclusions:**

Protein nm23 was not associated with a positiveness for tongue SCC without metastasis. Thus, several others factors inherent to host and malignancy can be associated with the mechanisms that suppress the metastatic process in this disease.

## INTRODUCTION

Head and neck carcinomas are the sixth most common cancer worldwide (INCA, 2006),^1^ the oral squamous cell carcinoma (OSCC) is the most frequent malignancy in this region. The prognosis of this entity in the mouth is poor; the local invasion and cervical lymph node metastasis rates are high.^2^, ^3^

Even with advances in oncological therapy, the five-year survival rate for patients with invasive OSCCs remains low (about 35%). Such morbidity rates may be due to factors such as varied responses to chemotherapy and radiotherapy, late presentation and delayed diagnosis. Although this malignancy has uniform morphological features, its biological behavior is heterogeneous, and tumors similarly staged may respond differently to the same treatment.^4^ Some authors have associated this clinical behavior with clinical staging (TNM) and anatomical site; those located in the tongue and floor of the mouth are considered more aggressive.^5^ Neck lymph node metastases and the TNM are the main measures of tumor aggressiveness, and are important parameters for planning the approach to such cases.

The metastatic process is complex; cells become dislodged from the primary tumor, the basement membrane and extracellular matrix degrade and are invaded, the tumor cells avoid the immune response in the blood circulation and eventually colonize distant sites.^6^ This process is how cancer progresses, and is frequent in OSCCs, especially those in the tongue and floor of the mouth, which explains the poor survival rate of patients with this disease.^7^

Although various metastasis-related events and proteins are known, certain aspects are poorly understood, such as why come tumors metastasize more frequently than others, as well as the identity of genes involved in this process. Identifying these genes may lead to the development of new strategies for the diagnosis and treatment of human cancers.

The nm23 gene appears to be involved in suppressing the metastatic process. It was first described by Steeg et al. in 1988, in experiments where this gene was transfected to mice melanomas. This gene is a family consisting of two genes in mice (nm23-1 and nm23-2) and six genes in humans, which code their respective proteins.^6^, ^8^ Decreased expression of its protein (nm23) in certain studies has been correlated with an increased metastasis rate and a poor prognosis in patients with breast, ovary, liver, stomach carcinomas and melanomas. This ratio, however, has not been demonstrated in neuroblastomas and bladder carcinomas.^9^ These findings remain unclear in OSCCs, as the few studies in this area have shown conflicting results.

More recently, a number of studies have investigated the relation between these genes and their expressed proteins with tumors in various anatomical sites; among these studies are those of Bertheau et al.,^10^ Fishman et al.,^11^ Kodera et al.,^12^ and Tokunaga et al.^13^ These studies have shown that nm23 protein expression is related with metastasis suppression in breast, thyroid, stomach and prostate carcinomas. Gunduz et al.^11^ found that the same applies to laryngeal squamous cell carcinomas; these authors suggested that the expression of this protein could be used as an indicator of the prognosis.

Improved therapy for OSCC aiming at reducing its metastatic potential and aggressiveness is necessary to increase the survival rate of patients with this disease. The purpose of this paper was to assess the immunohistochemical expression of the nm23-h1 protein in metastatic and non-metastatic squamous cell carcinomas of the tongue.

## MATERIAL AND METHOD

This retrospective study (a historical cohort) involved selecting 35 cases of squamous cell carcinomas of the tongue. Paraffin-included surgical specimens of patients from which the tumor had been resected and which had or had not done postoperative chemotherapy and/or radiotherapy were obtained. Sections 5 μm thick were made from these sections, which were then hematoxyllin-eosin stained for morphological analysis.

Cases with at least a 5-year follow-up were selected to investigate the presence of cervical lymph node metastases. Evidence for metastases in these cases was confirmed by image studies, such as computed tomography or magnetic resonance imaging, and/or morphological evidence in cervical dissection.

Sections 3 μm thick were made from the paraffin-included specimens, which were then streptavidin-biotin stained for immunohistochemistry; the sequence was as follows: deparaffinization; hydration in decreasing ethanol sequences; removal of the formolic pigment with 10% ammonium hydroxide in 95º ethanol; blocking of endogenous peroxidase by a 10 volume hydrogen peroxide solution (two 10’ steps), followed by antigen recovery in a 25’ steamer with the TRIS-EDTA solution (pH 9.0) and incubation of the anti-nm23-h1 monoclonal antibody, at 1:100 dilution, for a 60’ incubation time. The material was immersed in a TRIS pH 7.4 buffer solution between reaction steps. Next, it was incubated with a secondary antibody and the streptavidin-biotin complex during 30 minutes at room temperature; the reaction was developed with diaminobenzidine, followed by staining with Mayer's hematoxyllin and mounting on a Permount slide.

Breast ductal carcinoma sections were used as positive controls for verifying the effectiveness of the technique; incubation with the primary antibody was omitted in the negative control.

Two examiners in a double-blind study did the analysis of immune positivity; cases with brownish cytoplasmatic or nuclear staining were considered positive.

The Research Ethics Committee accepted this study (document number 72/03).

## RESULTS

The 35 cases of squamous cell carcinomas of the tongue consisted of 19 male cases (54.28%) and 16 female cases (45.71%); the mean age was 65 years. There were 22 patients that smoked cigarettes. Cervical lymph node metastases were found in 15 cases.

The tumor morphology was described as squamous cells with cellular and nuclear pleomorphism, nuclear hyperchromatism, prominent nucleoli, loss of the nuclear/cytoplasmatic ratio, typical and atypical mitoses, nests of varied sizes permeating loose or dense stromal connective tissue, and an inflammatory infiltrate with blood vessels.

**Figure 1:**
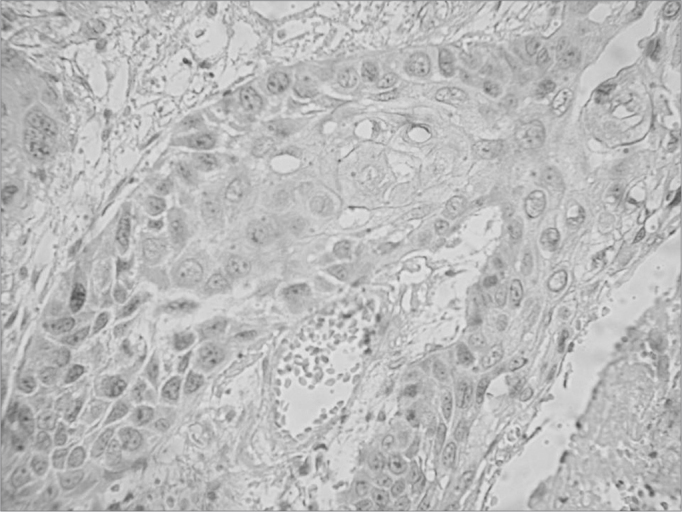
Immunohistochemical expression of the nm23-h1 protein in the cytoplasm of cells in the neoplastic squamous cell carcinoma of the tongue.

Immunohistochemically positive cells were those with a brownish color in the cytoplasm or nucleus (Figure 1) based on the aforementioned analysis.

Table 1 shows the results of the immunohistochemical analysis of the nm23 protein in our sample of squamous cell carcinomas of the tongue.

**Table 1:** Comparison of the immunohistochemical analysis of the nm23 protein in metastatic and non-metastatic squamous cell carcinomas of the tongue

		With metastases	With no metastases	Total
	Absence	10	11	21
nm23 protein	(Group 1)	66.7%	55%	
	Diffuse marking	5	9	14
	(Group 2)	33.3%	45%	
Total		15	20	35

Fischer's exact test revealed no statistically significant difference (p=0.365) in the comparison of immunohistochemical nm23 protein marking in metastatic and non-metastatic squamous cell carcinomas of the tongue. Table 1 also shows that there was no positivity in 10 of 15 metastasized cases.

## DISCUSSION

According to De La Rosa et al.,15 the expression of nm23 gene coded proteins may be verified by using anti-nm23 antibodies, as was done in this study. We used the anti-nm23-h1 antibody in 35 cases of squamous cell carcinomas of the tongue to check whether protein expression occurred in cases of squamous cell carcinomas of the tongue with no metastases to regional lymph nodes. Those authors found that mRNA levels for both the nm23-h1 and the nm23-h2 were decreased in the MDA-MB435 metastatic cell line, compared to the non-metastatic line.

The option to specifically verify the immune expression of the nm23-h1 protein was based on Garzia et al.'s^16^ statement that the nm23-h1 gene was the first gene to be discovered as having an important role in suppressing metastases. This expression induces anchorage-dependent cell colonization, making it difficult for ells to migrate.

The molecular mechanism of non-metastatic modulation and well-differentiated nm23 phenotypes is poorly understood. Lombardi et al.^17^ and Postel et al.^18^ have stated that the nm23 gene and its proteins are expressed physiologically during cell growth and differentiation, and that this expression varies in tissues. It may be found in the nucleus and the cytoplasm, associated with microtubules, on the cell and/or mitochondrial surface. Immune marking in our study was evident both in the nucleus and the cytoplasm of neoplastic cells. Khan et al.^7^ suggested a further mechanism in which cell migration may be inhibited in OSCCs, even though metalloproteinase 2 and 9 levels remain unaltered.

Ohtsuki et al.^19^ undertook an immunohistochemical analysis of nm23 protein expression in paraffin-included specimens of 33 OSCC cases and found that there may be suppression of metastases in this condition. Song et al.^20^ also reached this conclusion in a study of head and neck squamous cell carcinomas. These authors found no correlation with protein expression and the survival rate of patients. Wang et al.^21^ studied 86 OSCC cases and showed that smoking alters the expression of nm23-h1, facilitating disease progression. This may also have occurred in our sample, as most of our cases were chronic cigarette smokers.

There are few published papers in the literature demonstrating the role of the nm23 protein in suppressing metastases in the mouth. We were motivated to undertake the current study because we found no paper in the world literature that used the nm23 protein only in squamous cell carcinomas of the tongue; most of the published studies had investigated adenocarcinomas.

Fischer's exact test revealed no statistical significance in the expression of the nm23-h1 protein between the metastatic and non-metastatic cases of squamous cell carcinomas of the tongue in our sample. As Table 1 shows, diffuse marking of the nm23-hq protein was more evident in cases with no metastases. A further point is that of 15 metastatic cases, 10 were not immune-positive. Our results are similar to those of Göhring et al.^22^ in their 2002 paper, where nm23-h1 protein positivity was unrelated to metastatic breast carcinomas. On the other hand, our results disagree with those of Nascimento et al.,^23^ who investigated benign and malignant salivary gland neoplasms and found that the presence of the nm23 protein in the nucleus may be a good indicator for predicting the metastatic potential of salivary gland malignancies.

## CONCLUSION

Although we found no statistical significance between squamous cell carcinomas of the tongue and expression of the nm23 protein, we cannot discard its protective role against metastases. Thus, further prospective studies with more uniform clinically staged groups of OCSS cases are needed to demonstrate the aforesaid role in the metastatic process.
